# What Are the Merits of Our Chinese Physicians?

**Published:** 1869-09-15

**Authors:** 


### *What are the Merits of our Chinese Physicians?*

The question is intended to imply what are the merits of the Chinese practice of medicine as compared with our own? Our answer is that the Chinese practice of medicine is to ours what the practice before the days of Harvey is to the present.

The superiority of the modern practice over the crude empiricism of the past is almost entirely due to our knowledge of anatomy, physiology, comparative anotomy, pathology and chemistry, and of these sciences the Chinese physicians are profoundly ignorant. Our information in regard to the profound ignorance of the Chinese in these sciences is gathered from the Rev. A. W. Loomis' article in the *Overland Monthly* entitled "*Medical Art in the Chinese Quarter;*" from the writing of Dr. J. G. Kerr, who has charge of the "Medical Missionary Society's Hospital," in Canton; from an article in the *American Journal of Medical Sciences*, entitled "*Remarks on the actual state of Medical Science in Japan*, by Alex. M. Vedder, M.D., late surgeon United States navy, physician to His Highness, the Prince of Nagato and Suwo; from the reports of the "Medical Missionary Society in China;" from a letter received not long since from Dr. A. O. Treat, Pekin, China; from conversation, a few days since, with our friend Dr. Salter, American Consul at Hankow and Kinkiang for the past seven years, and other sources.

Dr. Vedder tells us that "The Japanese system of medicine is essentially based upon the Chinese, and nearly all medical books are written in the square Chinese character, which is read by all professional men." So that what Dr. Vedder tells us of the practice of medicine among the Japanese, is equally true of the Chinese. He says: "As to the professional acquirements of the Japanese faculty, dissection not being at all practiced in Japan, and even correct plates of the human structure being seldom seen, their knowledge of anatomy is exceedingly imperfect; still they have native names for the viscera, the arteries, veins, nerves, lymphatics, and principal anatomical structures, though topographical anatomy is entirely unknown; in physiology they are entirely in the dark, knowing, for example, nothing of the sympathetic system of nerves, of histology or animal chemistry, and attributing to the *liver* very important moral qualties. The circulation, also, is but imperfectly understood, the physician feeling the pulse at both wrists, from an impression that each side of the body is supplied by a corresponding side of the heart, independent of the other.

"Very many affections are supposed to proceed from the presence of various living organisms and worms infesting the economy, and going under the general name of 'mushi.' I have seen drawings purporting to represent these terrible creatures, and certainly, were any existing, should deem them fully capable of producing all the mischief ascribed to them.

"Most of the medicines employed are of Chinese origin, and are exhibited in bulky powders, or decoctions of certain vegetables, and of most forbidding appearance and taste. Hygiene, the sister of proplylaxis, is indeed a sealed book to the Japanese. As regards obstetrics, the practice is, to a great extent, in the hands of midwives. The use of forceps is unknown.

Of operative surgery the Japanese are profoundly ignorant; they possess but few instruments, and those of very rude construction, but, had they the whole modern 'armamentarium,' the want of anatomical knowledge would prevent them from being of much use. In case of fracture, no apparatus whatever for retention is employed, nor any attempt made at reduction; leeches and plasters alone being used to reduce the tumefaction and mitigate pain; a crutch made at my suggestion for one of the Prince's officers has been regarded as a miracle of skill and ingenuity. The medical men attached to the Japanese Embassy to the United States, practiced most freely upon the credulity of members of the profession there, in their accounts of hospitals at Yeddo, and of surgical operations performed in Japan. I need scarcely say that all these accounts were gratuitous falsehoods, and that there is not such a thing as a native hospital existing in Japan."

Dr. J. G. Kerr, in speaking of the Chinese physicians, says: "They have, however, no knowledge of the structure of the human system, or of the functions of the various parts. They are also ignorant of the nature of disease, and, to a considerable extent, of the properties of remedies. They have theories on all these subjects which are very elaborate and complicated, but which are absolutely false in every particular.

"The department of surgery can scarcely be said to exist. Beyond the applications of caustics and plasters to tumors and ulcers, and of poultices to broken bones, they are entirely helpless. There is no man in all the empire who can give aid, in any case of accident or disease which requires manual or instrumental interference. They are, of course, entirely without surgical instruments, and all the apparatus which modern ingenuity has applied to the relief of injuries, deformities and diseases."

Says the Rev. Mr. Loomis, "Very few of the Doctors, whether learned or ignorant, who are not more or less under the influence of superstition, believing in the power of supernatural influences and evil spirits to produce diseases, or in the necessity of aid from the good spirits to combat the agency of the inimical spirits."

"So prevalent is the belief among the common people, that the gods are often consulted for information as to which physician shall be employed; also, many apply directly to the gods for medical advice—to the Chinese Æsculapius, or some other god—or to a clairvoyant physician." For a full list of their disgusting remedies, and for a fuller account of their ignorant, superstitious, deceptive and revolting practices, are they not written in No. 6, Vol. 2, of the *Overland Monthly*.

That the Chinese practice of medicine is a compound of ignorance, superstition, and a kind of spiritualism, is a patent fact to the scientific world. For instance, they prescribe among other scientific and efficient remedies, hair,—dandruff,—animal exuviæ,—paring of finger and toe nails of pregnant women,—the placenta,—dragon's bones,—hoof of a white horse,—bull's manure,—ram's horns,—ears,—and many other things of like delicate nature.

REV. F. F. ELLINWOOD, D.D.

The tendency of the popular mind to run after the miraculous in medicine, is peculiar to no age.

"In physic as in fashion we find
The newest has always the run of mankind."

The more absurd the method, so long as it is new, the better. A mountebank professes to cure all ailments by the laying on of hands, and the people show their love of the marvelous, by flocking to him in great numbers, and the miracles of our Savior are put in the shade by the works of this new apostle. An imposter from the Celestial kingdom is the latest novelty in the healing persuasion; without respectability even among his own countrymen, "plenty good for Americans but no good for Chinamen," as an intelligent Chinese merchant remarked of this imposter, whom this brother imposter of a D.D., calls "one of the most skillful and most popular physician of San Francisco."

There is no doubt that this Chinaman's theology is equal to his medical knowledge; let us all go and worship at the Josh house. We hope Dr. Ellinwood will go and sit at the feet of their gods and learn something. There is no doubt but that he can learn more politeness and more christianity than he exhibits in this gratuitous insult to a learned profession.

When the Reverend gentleman is attacked again with gout or indigestion, which we hope will be soon, our best wish for him is, that he will find no better medical attendance than that this Chinese juggler can afford, and that he will give him no more palatable potion than the scrapings of his own toe nails, or a portion of his own ears. But he is the last man to apply for such skill; he will call on the best medical man he can find, probably in the middle of the night, and claim exemption from remunerating him, on the ground of his sacred calling.

This miserable fellow diagnoses all diseases as of one nature—promises cures to all—but wants his fees in advance. Persons in the last stages of consumption, when but few days are left to them on earth, are told that they have nothing but "bad liver and much kidney," and commit themselves to celestial aid to pass the Celestial Gate.

An educated physician is expected to cure everybody; he gets no credit for his cures, but his unsuccessful cases are scored up against him; but the Indian doctor, the spiritualist, and the Chinese barbarian, are not expected to cure, and their failings are passed in silence, but if some lucky one gets well in spite of them, it is trumpeted through the town as an astonishing cure. We set out to speak of Rev. F. F. Ellinwood, Doctor of Divinity, who used the sentence above quoted, in an article on the Chinese, in the *Occident*, of September 18th, but we set upon the other fellow; however, it makes no difference, they are a pretty pair. *Par ignobile fratrum.—California Paper.*

